# Nitric oxide-mediated modulation of the murine locomotor network

**DOI:** 10.1152/jn.00378.2013

**Published:** 2013-11-20

**Authors:** Joshua D. Foster, Catherine Dunford, Keith T. Sillar, Gareth B. Miles

**Affiliations:** School of Psychology and Neuroscience, University of St Andrews, St Andrews, Fife, United Kingdom

**Keywords:** spinal cord, neuromodulation, central pattern generator, motor control

## Abstract

Spinal motor control networks are regulated by neuromodulatory systems to allow adaptability of movements. The present study aimed to elucidate the role of nitric oxide (NO) in the modulation of mammalian spinal locomotor networks. This was investigated with isolated spinal cord preparations from neonatal mice in which rhythmic locomotor-related activity was induced pharmacologically. Bath application of the NO donor diethylamine NONOate (DEA/NO) decreased the frequency and modulated the amplitude of locomotor-related activity recorded from ventral roots. Removal of endogenous NO with coapplication of a NO scavenger (PTIO) and a nitric oxide synthase (NOS) blocker [nitro-l-arginine methyl ester (l-NAME)] increased the frequency and decreased the amplitude of locomotor-related activity. This demonstrates that endogenously derived NO can modulate both the timing and intensity of locomotor-related activity. The effects of DEA/NO were mimicked by the cGMP analog 8-bromo-cGMP. In addition, the soluble guanylyl cyclase (sGC) inhibitor ODQ blocked the effects of DEA/NO on burst amplitude and frequency, although the frequency effect was only blocked at low concentrations of DEA/NO. This suggests that NO-mediated modulation involves cGMP-dependent pathways. Sources of NO were studied within the lumbar spinal cord during postnatal development (postnatal days 1–12) with NADPH-diaphorase staining. NOS-positive cells in the ventral horn exhibited a rostrocaudal gradient, with more cells in rostral segments. The number of NOS-positive cells was also found to increase during postnatal development. In summary, we have shown that NO, derived from sources within the mammalian spinal cord, modulates the output of spinal motor networks and is therefore likely to contribute to the fine-tuning of locomotor behavior.

although at its core vertebrate locomotion involves rhythmic, stereotyped contractions of muscles, locomotor behavior must also be flexible so that it can be adjusted to suit changing environmental and developmental demands. The ability to produce rhythmic activity needed for locomotion is an inherent property of the spinal cord. The spinal motor network that produces this rhythm is termed the locomotor central pattern generator (CPG). The locomotor CPG is in turn regulated by a range of neuromodulatory systems that enable locomotor output to be adaptable (Miles and Sillar 2011). These modulatory inputs to the locomotor CPG can originate from either extrinsic sources, such as noradrenergic and serotonergic systems of the brain stem (as reviewed by Heckman et al. 2009; Rekling et al. 2000), or intrinsic sources within the spinal cord, for example, those utilizing adenosine (Witts et al. 2012), acetylcholine (Zagoraiou et al. 2009), and nitric oxide (NO) (Kyriakatos et al. 2009).

The gaseous neurotransmitter NO is a lipophilic free radical molecule with a short life span that is released when l-arginine is oxidized to l-citrulline by the enzyme nitric oxide synthase (NOS). Three isoforms of NOS have been identified (NOS1, nNOS; NOS2, iNOS; NOS3, eNOS), with the function of two of these variants, NOS1 and NOS3, being activity dependent because of their requirement for calcium/calmodulin binding. NO typically acts via the stimulation of soluble guanylyl cyclase (sGC) to increase intracellular levels of cGMP, which can act upon a range of intracellular and membrane-bound proteins (Ahern et al. 2002). However, additional mechanisms of action for NO exist, including *S*-nitrosylation of proteins and the generation of reactive oxygen species. It was first shown that NO produced by the endothelium, via NOS3, is essential for maintaining vascular tone (Huang et al. 1995; Shesely et al. 1996). It has also been shown that NOS3 activity provides a basal level of NO needed for the generation of hippocampal long-term potentiation (LTP), which is mediated by the additional release and summation of neuronally derived NO (O'Dell et al. 1994; Son et al. 1996). Furthermore, since NO can freely diffuse across biological membranes it can act as a volume transmitter to provide slow modulatory signals to both active and inactive neurons across whole neuronal networks (Steinert et al. 2008).

Previous investigations have shown that NO-mediated signaling has a marked neuromodulatory effect on spinal motor networks that generate the axial-based swimming movements of both *Xenopus* tadpoles (McLean and Sillar 2000, 2004) and lampreys (Kyriakatos et al. 2009; Kyriakatos and El Manira 2007). In the *Xenopus* tadpole, NO synthesized by a discrete cluster of neurons within the brain stem acts as an inhibitory extrinsic neuromodulator reducing the cycle frequency of swimming activity (McLean and Sillar 2000). During later developmental stages in *Xenopus*, when limbs and presumably their spinal control circuitry develop, NOS-expressing cells are also present in the spinal cord (Ramanathan et al. 2006). At these later stages NO may act as an intrinsic modulator of locomotion that, in contrast to early stages, increases the frequency of locomotor activity (Sillar et al. 2008). Several classes of spinal neurons in the adult lamprey have been demonstrated to express NOS (Kyriakatos et al. 2009). In lamprey spinal cord preparations, NO donors potentiate locomotor frequency while NOS inhibitors/NO scavengers decrease the frequency of locomotion, demonstrating an endogenous role for NO (Kyriakatos et al. 2009). In addition, NOS inhibitors occlude the LTP of locomotor frequency induced by activation of metabotropic glutamate receptors (Kyriakatos and El Manira 2007).

While the role of NO in the spinal locomotor circuitry of aquatic vertebrates is relatively well-defined, the role of NO in the control of mammalian locomotion has not previously been investigated despite evidence of considerable sources of NO in the mammalian spinal cord (Blottner and Baumgarten 1992; Burnett et al. 1995; Dun et al. 1993; Spike et al. 1993; Valtschanoff et al. 1992; Wetts et al. 1995). A modulatory role for NO has, however, been demonstrated for another rhythmic motor behavior in mammals, respiration, which is controlled by a brain stem CPG network. In rodents, NO donors appear to reduce the frequency and amplitude of spontaneous respiratory motor output while NOS inhibitors produce the opposite effect, indicating neuromodulation by endogenous NO (Mironov and Langohr 2007; Pierrefiche et al. 2007). These findings demonstrate that NO can modulate rhythmic motor activity produced by a mammalian CPG network. Our study therefore aimed to investigate whether NO plays a modulatory role within the mammalian spinal locomotor network, as has been shown for aquatic vertebrates. We demonstrate that NO, derived from sources within the spinal cord, modulates the output of spinal locomotor control circuitry in mice. Furthermore, we identify potential sources of this intrinsic, neuromodulatory NO and describe their postnatal development.

## METHODS

### 

#### Tissue preparation.

The use of animals in this investigation was in accordance with the United Kingdom Animals (in Scientific Procedures) Act 1986 and was reviewed and approved by the University of St Andrews Animal Welfare and Ethics Committee. For physiological preparations spinal cord tissue was prepared from postnatal day (P)2–P5 C57BL/6 mice, as described previously (Jiang et al. 1999). In brief, animals were killed by cervical dislocation, decapitated, and eviscerated before a section of the spinal cord extending from the midthoracic to upper sacral levels was isolated in a chamber containing artificial cerebrospinal fluid (aCSF; equilibrated with 95% O_2_-5% CO_2_, at room temperature). Isolated tissues were pinned down and the dorsal and ventral roots trimmed. Prepared tissues were then transferred to a recording chamber superfused with aCSF with a flow rate of 5–8 ml/min. For histological analyses spinal cord tissue was prepared from P1–P12 C57BL/6 or CD1 mice, as described above for physiological preparations, and tissues were then fixed via submersion in 4% paraformaldehyde (wt/vol) in 0.1 M phosphate buffer (PB; pH 7.4) for 12 h. The aCSF used for both dissections and recordings contained (in mM) 127 NaCl, 26 NaHCO_3_, 10 glucose, 3 KCl, 2 CaCl, 1.25 NaH_2_PO_4_, and 1 MgCl_2_.

#### Ventral root recordings.

Glass suction electrodes were attached to the first or second lumbar ventral roots (L_1_/L_2_), to record flexor-related activity, on both left and right sides at identical segmental levels of isolated spinal cord preparations (obtained from P2–P5 C57BL/6 mice). In a subset of experiments, extensor-related activity was also recorded from the fifth lumbar ventral root (L_5_). Rhythmic locomotor-related activity, alternating between contralateral upper lumbar roots and ipsilateral L_2_ and L_5_ roots, was induced pharmacologically with a combination of *N*-methyl-d-aspartic acid (NMDA, 5 μM) and 5-hydroxytryptamine (5-HT, 10 μM), with or without dopamine (50 μM). The inclusion of dopamine was associated with lower burst frequencies in control (with dopamine: 0.192 ± 0.019 Hz, *n* = 17; without dopamine: 0.246 ± 0.011 Hz, *n* = 38; Student's *t*-test, *P* < 0.05). However, statistical analyses demonstrated that the effects of subsequent drug applications did not differ between preparations with and without dopamine. For experiments investigating the importance of excitatory versus inhibitory components of the network we utilized a disinhibited preparation (Bracci et al. 1996) in which strychnine (1 μM) and either bicuculline (10 μM) or picrotoxin (60 μM) was added to block inhibitory transmission. This resulted in synchronous activity across contralateral roots. Activity recorded with bicuculline or picrotoxin did not differ; therefore preparations using either drug were grouped together for analyses [peak-to-peak amplitude: bicuculline 5.9 ± 0.9 a.b. (*n* = 16), picrotoxin: 6.2 ± 1.0 a.b. (*n* = 14), Student's *t*-test, *P* > 0.05; root-mean-squared (RMS) amplitude: bicuculline 0.48 ± 0.07 a.b. (*n* = 16), picrotoxin: 0.50 ± 0.08 a.b. (*n* = 14), Student's *t*-test, *P* > 0.05; frequency: bicuculline 0.061 ± 0.004 Hz (*n* = 16), picrotoxin: 0.054 ± 0.004 Hz (*n* = 14), Student's *t*-test, *P* > 0.05]. Subsequent drug applications were made once the bursts of locomotor-related activity recorded from ventral roots had stabilized (∼1 h; Witts et al. 2012). Drug washout was achieved via perfusion with drug-free aCSF. Data were amplified and filtered (band-pass filter 30–3,000 Hz; Qjin Design) and acquired at a sampling frequency of 6 kHz with a Digidata 1440A analog-to-digital converter and Axoscope software (Molecular Devices, Sunnyvale, CA). Custom-built amplifiers (Qjin Design) also allowed for the simultaneous online rectification and integration (50-ms time constant) of raw signals.

#### Data analysis.

Data were analyzed off-line with DataView software (courtesy of Dr. W. J. Heitler, University of St Andrews). Rectified/integrated traces were used for the detection of locomotor-related ventral root bursts. The instantaneous frequencies, peak-to-peak amplitudes, and RMS amplitudes (for disinhibited preparations) of ventral root bursts were then measured from raw traces. Amplitude was measured as a noncalibrated unit and is therefore presented as an arbitrary unit (a.b.). For time course plots data were aggregated into 1-min bins, or 2-min bins for disinhibited preparations, and normalized to a 10-min precontrol period to facilitate comparison between preparations. To calculate burst duration, individual bursts from a 10-min smoothed trace (4 iterations of 200 ms moving average) were downsampled to 100 Hz and the duration of each burst (the time taken from 40% peak amplitude on the upstroke to 40% peak amplitude on the downstroke) was calculated with a custom “R” script. Duty cycle was calculated as burst duration divided by cycle period. Statistical comparisons of burst amplitude and frequency were performed on absolute means, calculated over 5-min periods for standard preparations and 6-min periods for disinhibited preparations, for control, drug application, and washout phases. Data were analyzed with repeated-measures ANOVA and pairwise comparison with a Tukey's honestly significant difference (HSD) test, with *P* values < 0.05 taken as significant. Divergence of the data from sphericity was tested and Greenhouse-Giesser corrections applied where necessary. Repeated-measure ANOVAs and circular plot analyses were performed with “R” and the software packages “car” and “circular,” respectively. All data are presented as means ± SE.

The phase relationship between activity recorded from different pairs of ventral roots was assessed with circular plots and Rayleigh's test for uniformity (Kjaerulff and Kiehn 1996; Zagoraiou et al. 2009). Comparisons between the left-right and flexor-extensor phase relationships in control and drug conditions were made with the Watson-Williams test of homogeneity of means. In circular plots of left-right phase relationships, the “0” label denotes the timing of left ventral root bursts (at their peak amplitude) while individual data points denote the mean phase of right ventral root bursts (>100 bursts) in single preparations, with their distance from the center indicating the concentration of values around the mean (mean resultant vector). For circular plots of flexor-extensor phase relationships, the “0” label denotes the timing of flexor-related (L_2_) ventral root bursts (at their peak amplitude) while the individual data points indicate the mean phase of ipsilateral extensor-related (L_5_) root bursts (at their center of mass, >70 bursts). Center of mass, defined as the time point at which the voltage reached half of its cumulative summed total, was chosen for the L_5_ bursts because of the elongated shape of their extensor-related activity. Arrows associated with the circular plots indicate the mean direction of the phase relationships across all preparations analyzed.

#### NADPH-diaphorase histochemistry and cell counting.

After fixation, lumbar segments (1st to 5th) were cryoprotected in 30% sucrose (wt/vol) in PB at 4°C for 12 h and then frozen and stored at −20°C until being sectioned. Tissues were cut into 10- to 15-μm transverse sections on a cryostat and mounted on glass slides. Slides were incubated at 37°C with 1 mg/ml β-NADPH, 0.13 mg/ml nitro blue tetrazolium (NBT), and 0.3% Triton X-100 (vol/vol) in 0.1 M phosphate-buffered saline (PBS; pH 7.4) for 110 min and then washed in PBS to halt the reaction. In the presence of NOS and NADPH, NBT is reduced to the alcohol-insoluble blue dye nitro blue tetrazolium formazan, thereby indirectly labeling NOS-expressing cells for visualization under light microscopy (McLean and Sillar 2000).

Animals were categorized by age into four equally spaced groups (P1–P3, P4–P6, P7–P9, and P10–P12). With reference to Rexed's laminae, the dorsal horn was defined as lamina I–VI, the ventral horn as lamina VII–IX, and the area proximal to the central canal as lamina X. For each animal, images of five nonconsecutive (separated by at least 30 μm) spinal cord slices were obtained for each lumbar segment of interest. The number of positively stained cells in the dorsal horn, lamina X, and ventral horn were then counted manually. Although both C57BL/6 (*n* = 3) and CD1 (*n* = 24) mouse strains were used for histological analyses, no differences in the distribution of NADPH-diaphorase-positive cells were observed between the two strains. Given that the density of spinal neurons reduces developmentally, we adjusted cell counts to account for changes in lumbar spinal cord volume with age. The volume of the lumbar spinal cord was calculated from measurements of fixed spinal cord tissue (P1–P3, *n* = 5; P4–P6, *n* = 7; P7–P9, *n* = 6; P10–P12, *n* = 6). Lumbar volume estimates were calculated as the volume of a cylinder using the mean spinal cord diameter at L_1_, L_3_, and L_5_ and the mean length between L_1_ and L_5_ (P1–P3: 6.36 ± 0.45 mm^3^; P4–P6, 6.96 ± 0.35 mm^3^; P7–P9, 8.30 ± 0.12 mm^3^; P10–P12, 9.52 ± 0.13 mm^3^). With “R” and the package “MASS,” count data were fitted with a negative binomial generalized linear model using age and lumbar position as factors and log volume as an offset term (thereby fitting the model to counts per mm^3^ of volume). The influence of the factors age and lumbar position were assessed with likelihood ratio tests.

#### Immunohistochemistry.

Two days prior to experimentation CD1 mice received an intraperitoneal injection of Fluoro-Gold [40 mg/kg hydroxystilbamidine (Fluoro-Gold); Fluorochrome, Denver, CO] to retrogradely label sympathetic preganglionic neurons (SPNs) (Merchenthaler 1991). Spinal cords from these mice were dissected and fixed for 2 h, as described above, and stored in PBS at 4°C until being sectioned. Lumbar spinal slices at the L_1_–L_2_ level were cut at a thickness of 50 μm on a vibratome, and free-floating sections were incubated for 24 h at 4°C with rabbit primary antibodies raised against NOS1 (5 μg/ml; SAB43000426; Sigma-Aldrich, Poole, UK) in PBS with 1% bovine serum albumin (wt/vol) and 0.1% Triton X-100 (vol/vol). Bound primary antibody was labeled with fluorescein isothiocyanate (FITC)-tagged donkey anti-rabbit secondary antibody (3 μg/ml; 711-095-152; Jackson Immuno Research Laboratories, West Grove, PA) during a 2-h incubation. *Z*-plane optical sections of the stained tissue were created with an epifluorescence microscope and structured illumination (Imager.M2 fitted with Apotome.2, Carl Zeiss Microscopy, Göttingen, Germany), and the images were processed into a maximum *Z* projection (ImageJ; National Institutes of Health, Bethesda, MD).

#### Drug and solution preparation.

The NO donor diethylamine NONOate (DEA/NO) was supplied by Cambridge Bioscience (Cambridge, UK). The cGMP-dependent protein kinase activator 8-bromo-cGMP was obtained from Tocris Bioscience (Bristol, UK). The NOS inhibitor nitro-l-arginine methyl ester (l-NAME), the sGC inhibitor 1*H*-[1,2,4]oxadiazolo[4,3-*a*]quinoxalin-1-one (ODQ), and 5-HT were obtained from Abcam (Cambridge, UK). All other reagents were of analytical grade and supplied by either Fisher Scientific UK (Loughborough, UK) or Sigma-Aldrich. All drugs were dissolved in reverse osmosis water, except 2-phenyl-4,4,5,5-tetramethylimidazoline-1-oxyl 3-oxide (PTIO) and ODQ, which were dissolved in DMSO, and stored in aliquots at −20°C. Final concentration of DMSO in the working solutions was <0.1% (vol/vol).

## RESULTS

### 

#### Nitric oxide-mediated signaling modulates spinal locomotor circuitry.

**Fig. 1.**
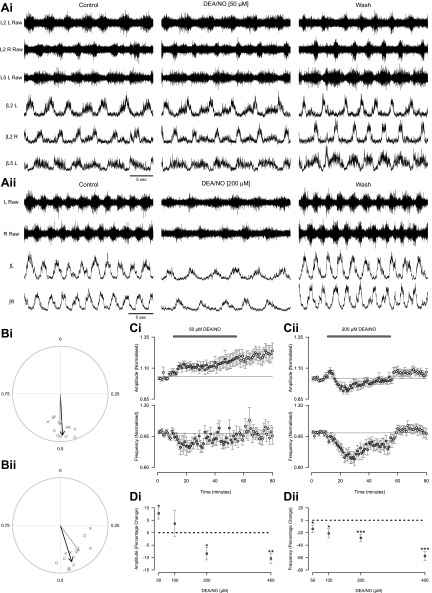
The nitric oxide (NO) donor diethylamine NONOate (DEA/NO) decreases the frequency and modulates the amplitude of locomotor-related activity in a concentration-dependent manner. *A*: raw (*top*) and rectified/integrated (*bottom*) traces recorded from left (L) and right (R) L_2_ ventral roots and the left (L) L_5_ ventral root (*Ai*) or both L_2_ ventral roots without L5 (*Aii*), showing the effects of 50 μM (*Ai*) and 200 μM (*Aii*) DEA/NO. *B*: circular plots displaying mean left-right phase relationships (*Bi*) in individual preparations (>100 bursts for each L_2_ root; 200 μM DEA/NO) or flexor-extensor phase relationships (*Bii*; >70 bursts for each root; 50–200 μM DEA/NO), with circles denoting measurements during control periods and crosses denoting those during DEA/NO application (*n* = 6 preparations). No change was identified between left-right or flexor-extensor phasing in control and drug conditions (*P* > 0.05). *Ci*: pooled time course plot of normalized data aggregated into 1-min bins shows an increase in amplitude (*top*) and a decrease in frequency (*bottom*) during 50 μM DEA/NO application (*n* = 15). *Cii*: time course plot during application of 200 μM DEA/NO shows a decrease in both normalized amplitude (*top*) and frequency (*bottom*) (*n* = 17). *D*: % change in locomotor burst amplitude (*Di*) and frequency (*Dii*), in response to varying concentrations of DEA/NO, calculated by comparing the last 5 min of control with a 5-min period during DEA/NO applications of varying concentrations. Statistically significant difference from control period: *P < 0.05, **P < 0.01, ***P < 0.001; *n* = 10–17.

To investigate whether NO-mediated signaling can modulate the activity of spinal motor networks controlling mammalian locomotion, we bath applied the NO donor DEA/NO at a range of concentrations (50 μM, *n* = 15 preparations; 100 μM, *n* = 13; 200 μM, *n* = 17; 400 μM, *n* = 10) to isolated spinal cord preparations while recording pharmacologically induced fictive locomotor activity from ventral roots (Fig. 1*A*). At all concentrations investigated, DEA/NO caused a reversible decrease in the frequency of bursts of locomotor-related ventral root activity (13.9 ± 6.1% decrease with 50 μM, 21.1 ± 7.5% decrease with 100 μM, 28.4 ± 6.0% decrease with 200 μM, and 57.6 ± 7.3% decrease with 400 μM; Fig. 1, *Ai*, *Aii*, *Ci*, *Cii*, and *Dii*). The modulatory effects of DEA/NO on the frequency of the locomotor rhythm were concentration dependent (Fig. 1*Dii*; DEA/NO concentration: *F*[3,50] = 7.9, *P* < 0.001; control/drug/wash: *F*[2,100] = 4.9, *P* < 0.01; interaction: *F*[6,100] = 4.7, *P* < 0.001, *n* = 10–17), with a maximum reduction observed at a dose of 400 μM. Given the variability in control frequencies of different preparations, we investigated whether there was a relationship between the starting frequency and the magnitude of the effect induced by DEA/NO; however, no significant relationship was identified.

In a subset of experiments we also assessed the effects of DEA/NO on burst duration and duty cycle. Burst duration was unchanged at all concentrations of DEA/NO (pooled mean for all DEA/NO concentrations: control 1.74 ± 0.04 s, DEA/NO 1.99 ± 0.09 s, washout 1.78 ± 0.08 s, *n* = 32 pooled; DEA/NO concentration: *F*[3,28] = 1.4, *P* > 0.05; control/drug/wash: *F*[2,56] = 1.2, *P* > 0.05; interaction: *F*[6,56] = 0.1, *P* > 0.05, *n* = 7–10 per DEA/NO concentration). Duty cycle was found to decrease with 200 μM and 400 μM DEA/NO (19.8 ± 6.7% decrease with 200 μM and 29.3 ± 5.2% decrease with 400 μM; DEA/NO concentration: *F*[3,28] = 4.5, *P* < 0.05; control/drug/wash: *F*[2,56] = 0.6, *P* > 0.05; interaction: *F*[6,56] = 4.6, *P* < 0.001, *n* = 7–10), but no significant change was observed with lower concentrations (50 μM, *n* = 7; 100 μM, *n* = 10).

DEA/NO also induced a concentration-dependent change in the amplitude of locomotor-related ventral root bursts (Fig. 1*Di*; DEA/NO concentration: *F*[3,50] = 3.5, *P* < 0.05; control/drug/wash: *F*[2,100] = 4.5, *P* < 0.05; interaction: *F*[6,100] = 4.1, *P* < 0.001, *n* = 10–17). In contrast to the consistent reduction in burst frequency, the polarity of the effect of DEA/NO on burst amplitude was concentration dependent. At higher concentrations (200 and 400 μM) DEA/NO caused a reduction in burst amplitude (8.4 ± 2.5% decrease with 200 μM, 10.4 ± 2.1% decrease with 400 μM; Fig. 1, *Aii*, *Cii*, and *Di*). However, at lower concentrations the application of DEA/NO either had no effect (100 μM; Fig. 1*Di*) or led to an increase in burst amplitude (7.8 ± 2.3% increase with 50 μM; Fig. 1, *Ai*, *Ci*, and *Di*). While the reduction in burst amplitude induced by high concentrations of DEA/NO was reversible, the increase in burst amplitude induced by 50 μM DEA/NO remained after drug washout and persisted for the duration of stable recordings (up to 60 min after initiation of washout). Control experiments in which no drugs were applied (beyond those used to elicit locomotor activity) demonstrated that locomotor burst amplitude is typically stable over a similar time course (*n* = 7; data not shown).

While the frequency, amplitude, and duty cycle of locomotor-related activity were modulated by DEA/NO, mean phase relationships of left-right (contralateral upper lumbar roots) and flexor-extensor (L_2_ vs. L_5_ ipsilateral roots) alternation remained unaltered (left-right phase, control: 0.492 ± 0.004, DEA/NO: 0.503 ± 0.009, *n* = 6; flexor-extensor phase, control: 0.450 ± 0.029, DEA/NO: 0.399 ± 0.031, *n* = 6). This was demonstrated by circular phase plots in which the mean direction of left-right (>100 bursts analyzed in control and drug conditions; Fig. 1*Bi*; Watson-Williams test, *F*[1,10] = 0.2, *P* > 0.05, *n* = 6) and flexor-extensor (>70 bursts analyzed in control and drug conditions; Fig. 1*Bii*; Watson-Williams test, *F*[1,10] = 1.5, *P* > 0.05, *n* = 6) alternation was unchanged by the application of DEA/NO (50–200 μM DEA/NO). The mean resultant length of the vectors for both left-right and flexor-extensor relationships was found to decrease in the presence of DEA/NO (L_2_ left/right relationship: control 0.87, DEA/NO 0.65; L_2_/L_5_ relationship: control 0.80, DEA/NO 0.67), demonstrating an increase in the variability of the phase relationships. This reduction in the strength of phase relationships is likely to result from an increase in the variability of locomotor output as revealed by greater coefficients of variation for frequency during the application of DEA/NO at all concentrations tested [50 μM: 0.138 ± 0.007 (control) vs. 0.245 ± 0.027 (DEA/NO); 100 μM: 0.194 ± 0.032 (control) vs. 0.379 ± 0.048 (DEA/NO); 200 μM: 0.143 ± 0.020 (control) vs. 0.362 ± 0.057 (DEA/NO); 400 μM: 0.152 ± 0.040 (control) vs. 0.483 ± 0.055 (DEA/NO); DEA/NO concentration: *F*[3,28] = 4.2, *P* < 0.05; control/drug/wash: *F*[2,56] = 2.6, *P* > 0.05; interaction: *F*[6,56] = 5.9, *P* < 0.001, *n* = 7–10].

In summary, these data demonstrate that NO-mediated signaling can modulate both the frequency and intensity of locomotor-related output generated by spinal motor circuitry. Thus NO may have multiple cellular sites of action including rhythm-generating interneurons and motoneurons. Interestingly, at lower concentrations, NO induces longer-term enhancement of the amplitude of locomotor network output.

#### Endogenous nitric oxide modulates locomotor network activity.

**Fig. 2.**
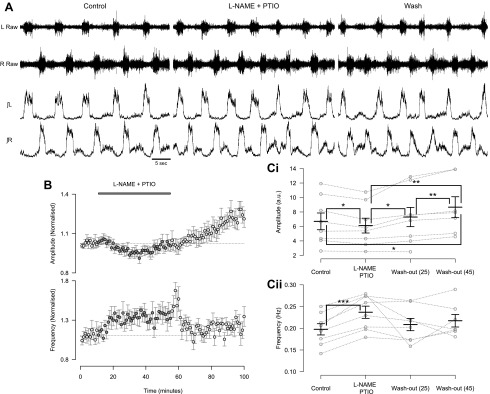
Removal of endogenous NO leads to a decrease in the amplitude and increase in the frequency of the locomotor-related activity. *A*: raw (*top*) and rectified/integrated (*bottom*) traces recorded from left and right L_2_ ventral roots showing the effect of coapplication of NOS inhibitor nitro-l-arginine methyl ester (l-NAME; 200 μM) and NO scavenger PTIO (400 μM). *B*: time course plots of normalized data aggregated into 1-min bins show a decrease in amplitude (*top*) and an increase in frequency (*bottom*) during coapplication of l-NAME and PTIO. *C*: locomotor burst amplitude (*Ci*) and frequency (*Cii*) during a 5-min period in control, l-NAME and PTIO application, and washout (25 and 45 min from start of drug application or washout). Individual data points are shown in gray, and mean is represented by black line. Statistically significant differences in pairwise comparisons: *P < 0.05, **P < 0.01, ***P < 0.001; *n* = 8. a.u., Arbitrary unit.

Next we investigated whether NO-mediated modulation represents an endogenous mechanism utilized by spinal cord circuitry to control locomotor output. To investigate the potential role of endogenous NO in the modulation of the murine locomotor network, we coapplied the NO scavenger PTIO (400 μM) and the NOS inhibitor l-NAME (200 μM) while recording fictive locomotion from isolated spinal cord preparations. When l-NAME and PTIO were coapplied to reduce the amount of endogenous NO available, we observed a decrease in the amplitude of locomotor-related ventral root bursts (7.7 ± 2.2%; Fig. 2, *A*, *B*, and *Ci*; *F*[3,18] = 11.8, *P* < 0.001, *n* = 8). Interestingly, the washout of l-NAME and PTIO was associated with an increase in amplitude during an extended washout period (18.7 ± 4.8% increase compared with control, *n* = 7). The similarity between this increase and the long-term effects of 50 μM DEA/NO application suggest that it may relate to a rebound in endogenous NO levels following the washout of blockers/scavengers. The coapplication of l-NAME and PTIO also led to an increase in the frequency (20.4 ± 2.4%; Fig. 2, *A*, *B*, and *Cii*; *F*[3,18] = 3.2, *P* < 0.05, *n* = 8) of locomotor-related activity. There was again no relationship between control frequency and the magnitude of frequency effects induced by l-NAME and PTIO. These findings demonstrate that NO is produced by the spinal cord during locomotor network activity. Furthermore, this endogenous NO modulates locomotor control circuitry to regulate the frequency and intensity of locomotor-related output.

#### Nitric oxide-mediated modulation involves the cGMP-dependent pathway.

**Fig. 3.**
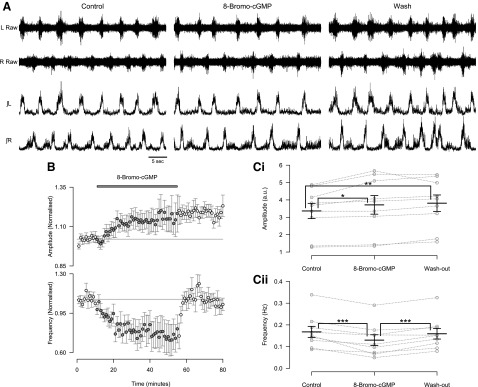
The cGMP analog 8-bromo-cGMP mimics the effects of DEA/NO on the frequency and amplitude of locomotor-related activity. *A*: raw (*top*) and rectified/integrated (*bottom*) traces recorded from left and right L_2_ ventral roots showing the effect of 8-bromo-cGMP (200 μM). *B*: time course plots of normalized data aggregated into 1-min bins show an increase in amplitude (*top*) and a decrease in frequency (*bottom*) during 8-bromo-cGMP application. *C*: locomotor burst amplitude (*Ci*) and frequency (*Cii*) during a 5-min period in control, 8-bromo-cGMP application (15 min into drug application), and washout (25 min from start of washout). Individual data points are shown in gray, and mean is represented by black line. Statistically significant differences in pairwise comparisons: *P < 0.05, **P < 0.01, ***P < 0.001; *n* = 9.

NO-mediated signaling typically involves the activation of NO-sensitive sGC and the subsequent accumulation of intracellular cGMP that can act on a range of intracellular and membrane-bound targets (Ahern et al. 2002). To elucidate the signaling pathways involved in NO-mediated modulation of spinal locomotor circuitry, we assessed the effects of pharmacological agents that target sGC-mediated signaling pathways. We first investigated the effects of application of a stable and membrane-permeant analog of cGMP, 8-bromo-cGMP (200 μM), on locomotor-related activity recorded from the isolated spinal cord preparation. The application of 8-bromo-cGMP led to a reversible reduction in the frequency of locomotor-related activity (24.9 ± 5.6%; Fig. 3, *A*, *B*, and *Cii*; *F*[2,16] = 16.3, *P* < 0.001, *n* = 9) and a sustained increase in the amplitude of locomotor-related ventral root bursts (8.9 ± 2.6%; Fig. 3, *A*, *B*, and *Ci*; *F*[2, 16] = 8.4, *P* < 0.01, *n* = 9). The actions of 8-bromo-cGMP mimic the effects of the lower concentration of DEA/NO (50 μM; Fig. 1, *Ai*, *Ci*, *Di*, and *Dii*) and support involvement of the sGC/cGMP pathway in the modulatory actions of NO on the murine locomotor control network.

**Fig. 4.**
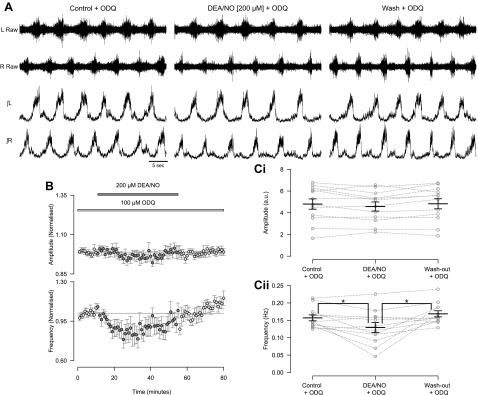
Inhibition of NO-sensitive soluble guanylyl cyclase (sGC) with ODQ blocks the effect of DEA/NO on the amplitude but not frequency of locomotor-related activity. *A*: raw (*top*) and rectified/integrated (*bottom*) traces recorded from left and right L_2_ ventral roots showing the effect of DEA/NO (200 μM) in the presence of ODQ (100 μM). *B*: time course plots of normalized data aggregated into 1-min bins show no change in amplitude (*top*) but a decrease in frequency (*bottom*) when DEA/NO is applied in the presence of ODQ. *C*: locomotor burst amplitude (*Ci*) and frequency (*Cii*) during a 5-min period in control with ODQ, DEA/NO application in the presence of ODQ (25 min into DEA/NO application), and washout with ODQ (25 min from start of washout). Individual data points are shown in gray, and mean is represented by black line. Statistically significant differences in pairwise comparisons: *P < 0.05; *n* = 12.

To further elucidate the role of the sGC/cGMP pathway we next applied DEA/NO (50 or 200 μM) in the presence of the sGC blocker ODQ (100 μM). The effects of both 50 and 200 μM DEA/NO on the amplitude of locomotor-related activity were blocked by ODQ (100 μM; DEA/NO 50 μM: *F*[2,12] = 1.8, *P* > 0.05, *n* = 7, data not shown; DEA/NO 200 μM: *F*[2,22] = 2.3, *P* > 0.05, *n* = 12, Fig. 4, *B* and *Ci*). ODQ also blocked the reduction in frequency induced by 50 μM (*F*[2,12] = 1.8, *P* > 0.05, *n* = 7; data not shown) but not 200 μM (Fig. 4, *A*, *B*, and *Cii*; *F*[2,22] = 7.9, *P* < 0.05, *n* = 12) DEA/NO. Comparison of the percent change in frequency induced by DEA/NO (200 μM) with and without ODQ (100 μM) showed no difference in the magnitude of this effect (Student's *t*-test, *P* > 0.05). At higher concentrations of ODQ (200 μM, *n* = 6; data not shown) the locomotor rhythm became unstable or ceased, so we could not test the possibility that 100 μM ODQ was an insufficient concentration to block the effects of 200 μM DEA/NO on locomotor frequency. Together these findings further support the idea that NO-mediated modulation of the intensity of locomotor output generated by spinal CPG circuitry involves the sGC/cGMP pathway.

#### NO-mediated modulation involves both excitatory and inhibitory components of the locomotor CPG.

**Fig. 5.**
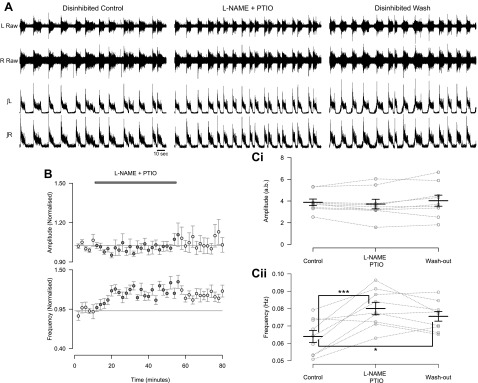
During the blockade of inhibitory neurotransmission the removal of endogenous NO leads to an increase in the frequency, but no change in the amplitude, of locomotor-related activity. *A*: raw (*top*) and rectified/integrated (*bottom*) traces recorded from left and right L_2_ ventral roots showing the effect of l-NAME (200 μM) and PTIO (400 μM) in disinhibited preparations in which inhibitory neurotransmission mediated by GABA (10 μM bicuculline or 60 μM picrotoxin) and glycine (1 μM strychnine) has been blocked. *B*: time course plots of normalized data aggregated into 2-min bins show no change in amplitude (*top*) and an increase in frequency (*bottom*) with coapplication of l-NAME and PTIO during disinhibition. *C*: locomotor burst amplitude (*Ci*) and frequency (*Cii*) during a 6-min period in control, l-NAME and PTIO application (16 min into drug application), and washout (26 min from start of washout). Individual data points are shown in gray, and mean is represented by black line. Statistically significant differences in pairwise comparisons: ***P < 0.001; *n* = 9. a.b., Arbitrary unit.

**Fig. 6.**
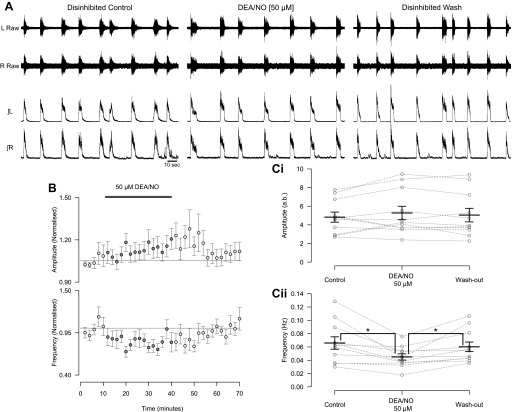
The NO donor DEA/NO can modulate both the amplitude and frequency of rhythmic activity during blockade of inhibitory neurotransmission. *A*: raw (*top*) and rectified/integrated (*bottom*) traces recorded from left and right L_2_ ventral roots showing the effect of 50 μM DEA/NO in disinhibited preparations in which inhibitory neurotransmission mediated by GABA (10 μM bicuculline or 60 μM picrotoxin) and glycine (1 μM strychnine) has been blocked. *B*: time course plots of normalized data aggregated into 2-min bins show a decrease in frequency during DEA/NO application during disinhibition. *C*: locomotor burst amplitude (*Ci*) and frequency (*Cii*) during a 6-min period in control, DEA/NO application (24 min into drug application), and washout (26 min from start of washout). Individual data points are shown in grey, and mean is represented by black line. Statistically significant differences in pairwise comparisons: *P < 0.05; *n* = 11.

To begin to explore the spinal components involved in NO-mediated modulation of locomotor control circuitry, we utilized disinhibited preparations (Bracci et al. 1996) to pharmacologically dissect out the effects of NO-mediated signaling on inhibitory and excitatory components of the network (Witts et al. 2012). Disinhibited preparations were produced by blocking inhibitory transmission with the glycine receptor antagonist strychnine (1 μM) and the GABA_A_ receptor antagonist bicuculline (10 μM) or the GABA_A_ channel blocker picrotoxin (60 μM). Ventral root recordings from these preparations revealed slow, rhythmic, large-amplitude bursts that were synchronous across segmentally aligned left and right ventral roots (Fig. 5*A* and Fig. 6*A*). In keeping with experiments performed in standard preparations, removal of endogenous NO, by coapplication of l-NAME and PTIO, in disinhibited preparations led to an increase in the frequency of locomotor-related ventral root bursts (26.7 ± 5.9%; Fig. 5, *A*, *B*, and *Cii*; *F*[2,16] = 11.8, *P* < 0.001, *n* = 9). However, l-NAME and PTIO had no effect on the peak-to-peak amplitude of locomotor-related bursts in disinhibited preparations (Fig. 5, *A*, *B*, and *Ci*; *F*[2,16] = 1.3, *P* > 0.05, *n* = 9). Given the irregular shape of disinhibited bursts, with a sharp peak followed by a lower-amplitude plateau, we also utilized RMS amplitude as a measure of the intensity of motor output. However, there was again no change in RMS amplitude with the application of l-NAME and PTIO (*F*[2,16] = 1.8, *P* > 0.05, *n* = 9). These findings indicate that endogenous NO modulates the frequency of locomotor activity via effects on excitatory components of the locomotor control network, while modulation of burst amplitude likely involves inhibitory components.

We next assessed the effects of exogenous NO on disinhibited preparations. The application of 50 μM DEA/NO to disinhibited preparations caused a decrease in the frequency of locomotor-related activity (26.5 ± 6.2%; Fig. 6, *A*, *B*, and *Cii*; *F*[2,20] = 8.4, *P* < 0.01, *n* = 11), and although there appeared to be a trend toward an increase in burst amplitude, this did not reach statistical significance whether measured as peak-to-peak or RMS amplitude (Fig. 6, *A*, *B*, and *Ci*; peak-to-peak: *F*[2,20] = 1.4, *P* > 0.05, *n* = 11; RMS: 5.1 ± 2.8%, *F*[2,20] = 3.4, *P* > 0.05, *n* = 11). In agreement with application of 50 μM DEA/NO, 200 and 400 μM DEA/NO also produced a decrease in the frequency of locomotor-related activity (200 μM: 27.5 ± 6.1%, *F*[2,12] = 15.2, *P* < 0.001, *n* = 7; 400 μM: 35.7 ± 11.5%, *F*[2,14] = 5.3, *P* < 0.05, *n* = 8; data not shown). However, in contrast to 50 μM DEA/NO, 200 and 400 μM DEA/NO produced a decrease in RMS burst amplitude (200 μM: 10.3 ± 2.0%, *F*[2,12] = 3.9, *P* < 0.05, *n* = 7; 400 μM: 20.5 ± 7.1%, *F*[2,14] = 6.1, *P* < 0.05, *n* = 8; data not shown) but no change in peak-to-peak amplitude (200 μM: *F*[2,12] = 0.2, *P* > 0.05, *n* = 7; 400 μM: *F*[2,14] = 3.7, *P* > 0.05, *n* = 8; data not shown).

For disinhibited preparations we again assessed whether the magnitude of the frequency effects induced by PTIO and l-NAME or DEA/NO were dependent on the starting frequencies of preparations. No such relationship was found for applications of l-NAME and PTIO. However, there was a linear relationship between the percent reduction in frequency induced by DEA/NO (50 μM) and the control frequency (β = −401.1, *P* > 0.05, *n* = 11), with slower initial burst frequencies associated with smaller DEA/NO-induced decreases in frequency. This may suggest there is a minimum frequency at which the already slow disinhibited preparations can generate rhythmic output.

These findings suggest that NO modulates the frequency of locomotor-related activity by affecting excitatory components of CPG circuitry. In contrast, the cellular components involved in NO-mediated modulation of the intensity of locomotor-related output appear to vary depending on the source and concentration of NO.

#### Location and development of NO sources within the murine spinal cord.

**Fig. 7.**
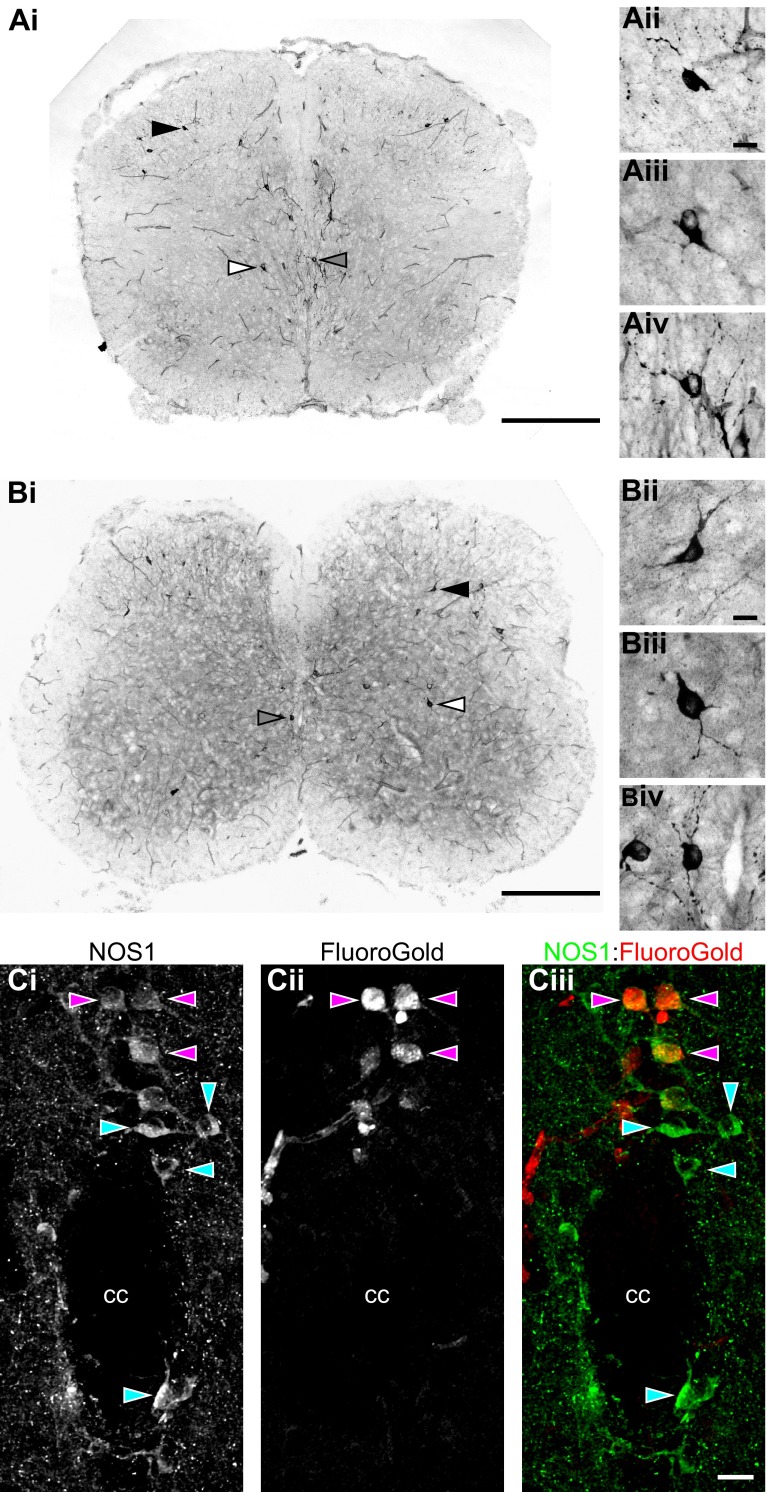
Sources of NO within the lumbar spinal cord labeled with NADPH-diaphorase staining. *A* and *B*: representative images of transverse spinal cord sections at the level of the 3rd segment (L_3_) taken from postnatal day (P)3 (*A*) and P10 (*B*) animals. Microvasculature and intensely stained cells are visible within the sections. The locations of cells shown in images *Aii–Aiv* and *Bii–Biv* (*insets*) are indicated with filled, open, and shaded arrowheads, respectively. *Insets* show positively stained cells in the dorsal horn (*ii*) and ventral horn (*iii*) and proximal to the central canal (*iv*). *C*: fluorescence images of a transverse spinal cord section (50-μm thickness, L_1_ level, P6 CD1 mouse) centered on the central canal (cc) showing immunohistochemical labeling using antibodies raised against NOS1 (*Ci*) and retrograde labeling of sympathetic preganglionic neurons (SPNs) with Fluoro-Gold (*Cii*). Merged image (*Ciii*) shows NOS1-positive SPNs (purple arrowheads) and non-SPN NOS1-positive cells (blue arrowheads). Scale bars: 500 μm in *Ai* and *Bi*; 20 μm in *Aii–Aiv, Bii–Biv*, and *Ci–Ciii*.

**Fig. 8.**
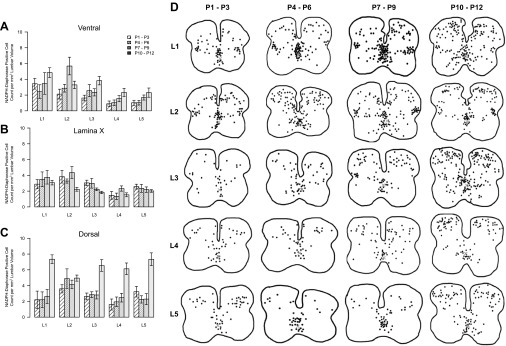
Distribution of NADPH-diaphorase-stained cells within the lumbar spinal cord. NADPH-diaphorase-positive cells were counted in the ventral horn (*A*), lamina X (*B*), and dorsal horn (*C*) of each lumbar segment (L_1_–L_5_) in animals across 4 different age groups (P1–P3, P4–P6, P7–P9, and P10–12; *n* = 6–9 animals per age group). In individual animals, cells were counted from 5 nonconsecutive sections from each lumbar segment. Counts were then averaged across animals and reported relative to lumbar spinal cord volume. *A*: distribution of NADPH-diaphorase-positive cells in the ventral horn displayed a rostrocaudal gradient, with more cells in rostral compared with caudal segments. In addition, the number of labeled cells in the ventral horn increased with age. *B*: NADPH-diaphorase-positive cells in lamina X also showed a rostrocaudal gradient, with a greater number of cells in rostral segments, while no significant developmental changes were observed. *C*: in the dorsal horn, NADPH-diaphorase-positive cells were evenly distributed along the rostrocaudal axis and increased in number with age. *D*: diagrams showing the positions of positively stained cells across 5 NADPH-diaphorase-stained sections at each lumbar segment (L_1_–L_5_) and across all age groups (P1–P3, P4–P6, P7–P9, and P10–P12). Sections and diagrams are orientated with the dorsal side at *top*, and the outlines represent the gray-white matter border.

Having established that endogenous NO derived from spinal sources modulates the activity of spinal locomotor circuitry, we next investigated the potential sources of this NO and their postnatal development. The distribution of NO sources within the lumbar spinal cord was elucidated by using NADPH-diaphorase staining to indirectly label cells expressing NOS over the postnatal developmental period P1–P12 (Fig. 7, *A* and *B*). This staining method has been previously shown to be selective for NOS-positive cells within the central nervous system (CNS) (Dawson et al. 1991; Hope et al. 1991). Staining was performed in transverse sections of lumbar spinal cord obtained from animals of four different postnatal age groups: P1–P3 (*n* = 6), P4–P6 (*n* = 6), P7–9 (*n* = 6), and P10–P12 (*n* = 9). At each of these age groups the number of NADPH-diaphorase-positive cells was assessed within the ventral horn, lamina X (proximal to the central canal), and the dorsal horn (Fig. 8) from the first to fifth lumbar segments (L_1_–L_5_). All cell counts were adjusted to the estimated lumbar volume for each age group.

NADPH-diaphorase-labeled neurons with intensely stained somata and proximal dendrites were found across all age groups and all lumbar segments (Fig. 7, *A* and *B*). Although the morphology and size of labeled cells varied, they were typically multipolar with somal diameters of ∼10–30 μm (Fig. 7, *A* and *B*). In addition to neuronal staining, the microvasculature was also observed to exhibit positive labeling at all lumbar levels. However, this vascular labeling was not quantified and was excluded from further analyses, which concentrated on neuronal sources of NO.

Perhaps most relevant to locomotor control, NADPH-diaphorase-positive neurons were consistently observed within the ventral horn of the lumbar spinal cord. Labeled cells were scattered throughout lamina VII and VIII but were rarely seen within motoneuron pools (lamina IX), with less than one cell per animal in lamina IX for each lumbar segment (Fig. 7, *A* and *B*, and Fig. 8*D*). Cell counts revealed a rostrocaudal gradient of NADPH-diaphorase-positive neurons in the ventral horn, with greater numbers of cells in rostral lumbar segments (χ^2^[1,*n* = 132 counts from 27 animals] = 36.4, *P* < 0.001). Analyses across different age groups also revealed an increase in NADPH-diaphorase-positive neurons in the ventral horn with age (χ^2^[1,*n* = 132 counts from 27 animals] = 18.8, *P* < 0.001; Fig. 7, *A* and *B*, and Fig. 8*A*). Given that counts of NADPH-diaphorase-positive neurons in lumbar segments 1 and 2 will include NOS-positive SPNs (Blottner and Baumgarten 1992; Burnett et al. 1995; Dun et al. 1993; Wetts et al. 1995), the rostrocaudal gradient of counted cells may relate to the localization of SPNs to upper lumbar segments. We therefore also analyzed cell counts from L_3_–L_5_, where SPNs are not present (Anderson et al. 1989; Burnett et al. 1995). The rostrocaudal gradient of NADPH-diaphorase-positive neurons remained across these segments (χ^2^[1,*n* = 78 counts from 27 animals] = 10.5, *P* < 0.01).

NADPH-diaphorase-positive cells were also found to cluster in lamina X around the central canal of the spinal cord (Fig. 7, *A* and *B*, and Fig. 8, *B* and *D*), an area that has also been implicated in locomotor control (Huang et al. 2000; Zagoraiou et al. 2009). In parallel with the ventral horn, the number of NADPH-diaphorase-positive cells in lamina X exhibited a rostrocaudal gradient, with more cells found in rostral segments (χ^2^[1,*n* = 132 counts from 27 animals] = 23.8, *P* < 0.001). However, in contrast to the ventral horn, the number of NADPH-diaphorase-positive cells in lamina X did not change with age (χ^2^[1,*n* = 132 counts from 27 animals] = 3.8, *P* > 0.05; Fig. 7, *A* and *B*, and Fig. 8*B*). The NADPH-diaphorase-positive neurons counted in lamina X will also include SPNs (Burnett et al. 1995; Dun et al. 1993; Wetts et al. 1995; Fig. 7*C*), which may account for the rostrocaudal gradient of labeled cells. To investigate this further, we therefore again analyzed lamina X cell counts from L_3_–L_5_. This analysis failed to reveal a rostrocaudal gradient of NADPH-diaphorase-positive neurons across L_3_–L_5_ (χ^2^[1,*n* = 78 counts from 27 animals] = 0.38, *P* > 0.05). Thus the gradient observed in lamina X along the entire lumbar cord reflects the presence of SPNs in upper lumbar segments. To demonstrate the presence of NOS-positive interneurons among SPNs in lamina X of the upper lumbar cord, we also performed double-fluorescence labeling experiments using Fluoro-Gold to retrogradely label SPNs and NOS1 immunohistochemistry to label nitrergic neurons. Consistent with previous reports (Dun et al. 1993; Miles et al. 2007; Wetts et al. 1995), we found a population of NOS1-positive neurons in lamina X of the upper lumbar spinal cord that are not Fluoro-Gold labeled and are therefore not SPNs (Fig. 7*C*).

Finally, as reported previously (Spike et al. 1993; Valtschanoff et al. 1992; Wetts et al. 1995), many NADPH-diaphorase-positive neurons were found within the dorsal horn of the lumbar spinal cord (laminae I–IX). Data from the different dorsal lamina were pooled together into a single group. The numbers of NADPH-diaphorase-positive cells in the dorsal horn did not differ between the lumbar segments (χ^2^[1,*n* = 132 counts from 27 animals] = 0.4, *P* > 0.05) but were found to increase with the age of animals (χ^2^[1,*n* = 132 counts from 27 animals] = 33.0, *P* < 0.001; Fig. 7, *A* and *B*, and Fig. 8*C*). At the earliest postnatal stages the number of NADPH-diaphorase-positive neurons in the dorsal horn was similar to that in the ventral horn or lamina X, but by P10 the dorsal horn contained the greatest concentration of NADPH-diaphorase-positive neurons in the lumbar spinal cord.

In summary, our anatomical analyses revealed considerable vascular and neuronal sources of NO within the lumbar spinal cord, including in ventral regions where the locomotor circuitry is thought to reside and in dorsal regions that may contribute to sensory-motor integration. Ventral NO sources exhibited a rostrocaudal bias and, along with dorsal sources, increased in number during postnatal development.

## DISCUSSION

The results of this study demonstrate that the free radical NO can modulate mammalian spinal locomotor control networks. Furthermore, we have shown that endogenous NO, derived from sources within the spinal cord, plays a role in setting the frequency and intensity of locomotor-related rhythmic activity generated by the mammalian spinal cord. These findings suggest that NO acts as an intrinsic neuromodulator of both interneurons within the locomotor CPG and motoneurons conveying the final motor output of the CNS (Miles and Sillar 2011).

The addition of exogenous NO, via the NO donor DEA/NO, to isolated mouse spinal cord preparations during stable pharmacologically induced locomotor activity was shown to consistently reduce the frequency of locomotor-related rhythmic activity in a concentration-dependent manner. This change in frequency was associated with no change in burst duration but a decrease in duty cycle at higher concentrations of DEA/NO. The lack of a significant change in duty cycle with low concentrations of DEA/NO may relate to the relatively small change in frequency these concentrations induce. Interestingly, constant L_2_ (flexor related) burst durations along with decreases in duty cycle may relate to whole animal studies showing that during changes in locomotor speed the flexor (swing) phase remains relatively constant while the extensor (stance) phase is altered (Pearson 1976).

In contrast to the effects on frequency, the polarity of the effects of exogenous NO on the amplitude of locomotor-related activity was concentration dependent, with lower concentrations of DEA/NO increasing and high concentrations decreasing the amplitude of locomotor-related output. These results are analogous to previous studies showing opposing, concentration-dependent effects of exogenous NO on neurotransmitter release (Prast et al. 1998) and neuronal firing (Pierrefiche et al. 2007) and suggest that NO-mediated signaling might in some circumstances change the nature of motor output rather than simply fine-tuning it. Given reports that reductions in locomotor frequency are associated with increases in the amplitude of locomotor-related output (e.g., Barriere et al. 2004), it might be argued that the increase in amplitude we observed with low concentration of DEA/NO reflects some underlying inverse correlation between these two parameters. In opposition to this, we report a sustained increase in amplitude despite the locomotor frequency returning to control levels following drug washout (Fig. 1*Ci*). Furthermore, several studies have demonstrated that modulation of locomotor-related frequency does not necessarily coincide with changes in amplitude, and vice versa (e.g., Kiehn et al. 1999; Sqalli-Houssaini and Cazalets 2000; Witts et al. 2012).

Removal of endogenous NO, via the blockade of NOS and scavenging of NO, increased the frequency and decreased the amplitude of locomotor-related output. These data demonstrate that NO is not just a potential modulator of spinal motor circuitry but endogenously derived NO actually influences locomotor control circuitry during ongoing activity. Although analyses of NOS1 (Nelson et al. 1995)- and sGC (Schmidtko et al. 2008)-knockout mice have revealed no overt changes in gross motor activity, it would be interesting to investigate potential deficits in their ability to modulate motor output (e.g., swim vs. walking tasks; Zagoraiou et al. 2009). The recent creation of NOS1-Cre mouse lines (Taniguchi et al. 2011) may also facilitate more direct investigations of the roles of NOS1-positive interneurons in locomotor control. In our study the effects of blockade of NO-mediated signaling were found to be opposite to the effects of application of low concentrations of exogenous NO (50 μM DEA/NO). Thus lower concentrations of exogenous NO most likely demonstrate the endogenous, physiological roles of NO in motor control circuitry. In contrast, the effects of higher concentrations of the NO donor may represent nonphysiological or possibly pathophysiological conditions. Interestingly, increased expression of NOS isoforms has been implicated in pathological conditions such as amyotrophic lateral sclerosis (Catania et al. 2001; Phul et al. 2000) and arthritis-induced hyperalgesia (Wu et al. 1998).

The demonstration that NO acts as an intrinsic modulator of spinal locomotor control circuitry in mice parallels previous investigations of locomotor circuitry in aquatic vertebrates (Kyriakatos et al. 2009; Kyriakatos and El Manira 2007; McLean and Sillar 2000, 2004) and respiratory control circuitry in rodents (Mironov and Langohr 2007; Pierrefiche et al. 2007). Our findings are most similar to those of previous studies in the *Xenopus* tadpole, in which NO was found to act in an inhibitory manner, slowing the frequency of activity produced by the locomotor CPG (McLean and Sillar 2000). Interestingly, in the pro-metamorphic *Xenopus* (Sillar et al. 2008) and adult lamprey (Kyriakatos et al. 2009; Kyriakatos and El Manira 2007) the intrinsic neuromodulatory effects of NO are opposite to those observed in the *Xenopus* tadpole, with NO-mediated signaling leading to an increase in the frequency of locomotor activity at later stages. These data support, at least in *Xenopus*, a developmental reversal in the role of NO in locomotor control that might relate to changes in locomotor function due to metamorphosis. Whether similar developmental changes might alter the nature of the modulatory effect of NO on mouse locomotor circuitry remains to be determined, although one might predict more subtle changes than those observed in the metamorphic *Xenopus*.

In the *Xenopus* tadpole the inhibitory effect of NO on locomotor frequency results largely from its facilitation of GABAergic and glycinergic transmission (McLean and Sillar 2002, 2004), while in the lamprey NO-mediated facilitation of locomotor frequency involves modulation of both inhibitory and excitatory transmission (Kyriakatos et al. 2009). Our data, demonstrating that NO-mediated modulation of the frequency of motor activity persists when inhibition is blocked, suggest that the effects of NO on motor activity generated by the mouse spinal cord involve modulation of excitatory rather than inhibitory components of spinal motor circuitry. In contrast, the effects of endogenous NO on the intensity of motor output generated by the mouse spinal cord may involve inhibitory components of the network, since blockade of NO-mediated signaling no longer affects the amplitude of motor bursts when inhibition is also blocked. Interestingly, exogenous NO can still modulate the amplitude of motor activity in the absence of inhibition. This suggests that the intensity of motor output can also be modulated by mechanisms involving inhibitory components of spinal motor circuitry but that under our experimental conditions such mechanisms are unlikely to be engaged by NO derived from spinal sources. When interpreting data from disinhibited preparations it should be noted that, although indirect evidence supports involvement of common motor circuitry in the generation of disinhibited bursting and fictive locomotor activity (Bracci et al. 1996; Kiehn 2006), it remains unknown whether the same populations of neurons underlie these two rhythmic outputs. Further analyses at the cellular level will therefore be required to elucidate the exact components of the spinal motor circuitry that are modulated by NO-mediated signaling.

The main target for NO is sGC, which is widely expressed throughout the mouse lumbar spinal cord (Schmidtko et al. 2008), and the subsequent activation of cGMP-dependent pathways. Utilizing a membrane-permeant cGMP analog, 8-bromo-cGMP, we have demonstrated that the sGC system can modulate both the amplitude and frequency of locomotor-related output generated by the mouse spinal cord. The effects of 8-bromo-cGMP mimic those of low concentrations of an NO donor (50 μM DEA/NO). In addition, inhibition of sGC with ODQ was found to block NO-mediated modulation of locomotor burst amplitude. These data are consistent with NO modulating motoneurons, and/or the synaptic inputs they receive, via sGC/cGMP-dependent pathways, as has previously been demonstrated in brain stem motoneurons (Abudara et al. 2002; Mironov and Langohr 2007; Montero et al. 2008; Wenker et al. 2012). In contrast, ODQ blocked the modulation of locomotor burst frequency mediated by low but not high concentrations of DEA/NO. These data suggest involvement of sGC/cGMP-dependent and potentially additional signaling pathways, such as those involving *S*-nitrosylation of proteins (Ahern et al. 2002), in NO-mediated modulation of rhythm-generating components of the murine locomotor circuitry. Although the concentration of ODQ used in this investigation is equal to or higher than that used in previous studies (Kyriakatos et al. 2009; Kyriakatos and El Manira 2007; Mironov and Langohr 2007; Pierrefiche et al. 2007), it is still possible that we were unable to fully block sGC with ODQ. However, higher concentrations of ODQ (up to 400 μM) could not be used because they were found to disrupt the pharmacologically induced rhythm. This may further support the importance of NO-mediated signaling in locomotor control, although the potential for off-target effects of ODQ at high concentrations cannot be discounted.

Along with acute effects on locomotor-related activity, applications of NO donor at lower concentrations (50 μM DEA/NO) or 8-bromo-cGMP to the isolated spinal cord led to a sustained increase in the amplitude of locomotor-related activity. These long-lasting effects, which extended into the washout period and remained as long as stable rhythms could be recorded, may represent an LTP-type effect of NO on the locomotor control network. Similar effects were also seen during an extended washout of l-NAME and PTIO, which may result from a rebound in endogenous NO levels following removal of blockers/scavengers. NO-mediated signaling is known to contribute to LTP of glutamatergic synapses in the hippocampus (Garthwaite and Boulton 1995; Prast and Philippu 2001) and, more recently, GABAergic synapses in lamina I of the spinal dorsal horn (Fenselau et al. 2011). In addition, and perhaps most relevant to the present study, NO is involved in the LTP of locomotor frequency evoked by activation of metabotropic glutamate receptor 1 in the lamprey spinal cord (Kyriakatos and El Manira 2007). Our findings, indicating an LTP-like effect on the amplitude of locomotor-related activity, may demonstrate a novel plastic effect of NO on components of the mammalian locomotor system. It will be important to investigate the cellular mechanisms of this plasticity in future studies.

In addition to examining the physiological effects of NO-mediated signaling on spinal motor circuitry, we also investigated spinal sources of NO and their postnatal development by using the NADPH-diaphorase labeling technique. The pattern of NOS-positive neurons demonstrated in this investigation are in agreement with those shown previously in the adult rat with NADPH-diaphorase histochemistry (Spike et al. 1993; Valtschanoff et al. 1992; Wetts et al. 1995) and NOS immunohistochemistry (Burnett et al. 1995; Dun et al. 1993). Given the localization of the locomotor CPG to the ventral horn of the spinal cord (Cazalets et al. 1995; Kjaerulff and Kiehn 1996), NOS-positive neurons found scattered throughout most of the ventral horn are well-positioned to contribute to NO-mediated modulation of locomotor activity. NOS-positive cells in lamina X surrounding the central canal of the spinal cord may also contribute, since neurons within this region appear to play a role in the control of locomotion (Huang et al. 2000; Zagoraiou et al. 2009). Previous investigations have demonstrated that NO donors alter the frequency of tonic-firing lamina X interneurons in slices from rat lumbar spinal cord (Pehl and Schmid 1997), further supporting the potential importance of NO production and release in this area. In addition, given the influence of NO-mediated signaling on sensory processing, particularly nociceptive (Freire et al. 2009), it is also possible that NOS-positive neurons within the dorsal horn influence locomotor output via pathways involved in sensory-motor integration.

In agreement with developmental findings in the rat lumbar spinal cord (Wetts et al. 1995), we found that the numbers of NOS-positive neurons within both the dorsal and ventral horns of the mouse spinal cord increase during postnatal development. In contrast, the number of positive cells surrounding the central canal remains relatively static during this period of development. Interestingly, the time course of this increase in the number of NO sources follows the time period over which mice progress from crawling to walking freely with the ventral surface of the body off the ground (approximately P10; Jiang et al. 1999). Our investigation also demonstrated a rostrocaudal bias in the distribution of the NOS-positive neurons in the ventral horn and lamina X of the lumbar spinal cord. These findings parallel the reported rostrocaudal bias of the locomotor CPG to upper lumbar segments (Cazalets et al. 1995; Kjaerulff and Kiehn 1996) and provide further, albeit indirect, evidence linking NOS-positive neurons to the locomotor CPG.

In agreement with previous studies (Dun et al. 1993; Spike et al. 1993; Valtschanoff et al. 1992), we find very few NO sources within or immediately adjacent to motoneuron pools. However, we have demonstrated that removal of intrinsic NO alters motor output in a manner consistent with modulation of motoneurons or last-order synaptic inputs they receive (Miles and Sillar 2011). Other physiological investigations have shown NO to alter the excitability of trigeminal (Abudara et al. 2002) and hypoglossal (Montero et al. 2008) motoneurons and modulate both inhibitory and excitatory synaptic inputs to the motoneurons (Abudara et al. 2002; Kyriakatos et al. 2009). While NO can act as a volume transmitter, with a sphere of influence based on diffusion in CNS tissue of ∼20 μm from its source (Steinert et al. 2008), the lack of labeled cell bodies within or near motoneuron pools may indicate that local interneurons are not the primary source of the NO affecting motoneurons. However, given that NO may be released from dendrites or NOS-positive synaptic terminals, which have been reported within motor pools (Miles et al. 2007), the influence of NOS-positive interneurons may be farther reaching than the location of their cell bodies suggests. Alternatively, in parallel with findings in the hippocampus, where basal levels of NO provided by the vasculature are known to be required for LTP (Hopper and Garthwaite 2006; O'Dell et al. 1994; Son et al. 1996), it is possible that the endogenous NO that modulates locomotor-related output arises from the microvasculature of the spinal cord. At present there is a lack of selective NOS3 inhibitors (Boer et al. 2000), limiting our ability to directly investigate the contribution of vascular sources of NO.

In summary, we have demonstrated that endogenous NO, derived from sources within the developing mouse spinal cord, modulates locomotor-related output produced by spinal motor circuitry. At concentrations we speculate are most physiologically relevant, NO-mediated modulation includes long-term changes to the intensity of locomotor-related output. Meanwhile, experiments investigating the effects of higher, possibly pathological, concentrations of NO highlight potentially deleterious inhibitory effects this modulator may have on motor output. While advancing our understanding of the neuromodulatory control of spinal motor networks, this study also provides a platform from which to investigate the influences of NO at the cellular level. Such work is not only important for further advancement of our basic understanding of motor control but may also highlight pathways that can be targeted for the treatment of injury and disease affecting the spinal cord.

## GRANTS

This work was supported by the Wellcome Trust (Grant no. 091177/Z/10/Z). C. Dunford was supported by a Biotechnology and Biological Sciences Research Council (BBSRC) studentship.

## DISCLOSURES

No conflicts of interest, financial or otherwise, are declared by the author(s).

## AUTHOR CONTRIBUTIONS

Author contributions: J.D.F., C.D., and G.B.M. conception and design of research; J.D.F. and C.D. performed experiments; J.D.F. and C.D. analyzed data; J.D.F., C.D., K.T.S., and G.B.M. interpreted results of experiments; J.D.F. and C.D. prepared figures; J.D.F. and G.B.M. drafted manuscript; J.D.F., C.D., K.T.S., and G.B.M. approved final version of manuscript; C.D., K.T.S., and G.B.M. edited and revised manuscript.
